# Differential requirement of the epidermal growth factor receptor for G protein-mediated activation of transcription factors by lysophosphatidic acid

**DOI:** 10.1186/1476-4598-9-8

**Published:** 2010-01-14

**Authors:** Regina A Oyesanya, Susie Greenbaum, David Dang, Zendra Lee, Abir Mukherjee, Jinhua Wu, Paul Dent, Xianjun Fang

**Affiliations:** 1 From the Departments of Biochemistry & Molecular Biology, Virginia Commonwealth University School of Medicine, 1101 East Marshall Street, Richmond, VA 23298, USA

## Abstract

**Background:**

The role of the epidermal growth factor receptor (EGFR) and other receptor tyrosine kinases (RTKs) in provoking biological actions of G protein-coupled receptors (GPCRs) has been one of the most disputed subjects in the field of GPCR signal transduction. The purpose of the current study is to identify EGFR-mediated mechanisms involved in activation of G protein cascades and the downstream transcription factors by lysophosphatidic acid (LPA).

**Results:**

In ovarian cancer cells highly responsive to LPA, activation of AP-1 by LPA was suppressed by inhibition of EGFR, an effect that could be reversed by co-stimulation of another receptor tyrosine kinase c-Met with hepatocyte growth factor, indicating that LPA-mediated activation of AP-1 requires activity of a RTK, not necessarily EGFR. Induction of AP-1 components by LPA lied downstream of Gi, G12/13, and Gq. Activation of the effectors of Gi, but not Gq or G12/13 was sensitive to inhibition of EGFR. In contrast, LPA stimulated another prominent transcription factor NF-κB via the Gq-PKC pathway in an EGFR-independent manner. Consistent with the importance of Gi-elicited signals in a plethora of biological processes, LPA-induced cytokine production, cell proliferation, migration and invasion require intact EGFR.

**Conclusions:**

An RTK activity is required for activation of the AP-1 transcription factor and other Gi-dependent cellular responses to LPA. In contrast, activation of G12/13, Gq and Gq-elicited NF-κB by LPA is independent of such an input. These results provide a novel insight into the role of RTK in GPCR signal transduction and biological functions.

## Background

Lysophosphatidic acid (LPA, 1-acyl-*sn*-glycerol-3-phosphate) is a naturally occurring intercellular mediator of diverse biological functions [1]. It is produced by activated platelets during coagulation and thus is a normal constituent of serum [2,3]. At least six G protein-coupled receptors (GPCRs) of LPA have been identified [4]. The LPA_1_/Edg2, LPA_2_/Edg4 and LPA_3_/Edg7 receptors are members of the endothelial differentiation gene (Edg) family and share 50-57% homology in their amino acid sequences [5-7]. Recently, LPA_4_/p2y9/GPR23, LPA_5_/GPR92 and LPA_6_/p2y5 of the purinergic receptor family, structurally distant from the Edg LPA receptors were described as additional LPA receptors [8-11]. The LPA receptors couple to multiple G proteins, G12/13, Gi, Gq, and probably Gs [4]. These G proteins link to diverse signaling pathways including stimulation of phospholipase C and D, inhibition of adenylyl cyclase, and activation of Ras and the downstream mitogen-activated protein kinases and phosphoinositide 3-kinase [4,12]. Activation of these signaling cascades downstream of LPA receptors culminates in morphological changes and promotion of cell growth, survival and motility. Recently, we and others demonstrated that LPA induces activation of various transcription factors, upregulating expression of many target genes involved in cell proliferation, survival, and migration and invasion [13-20]. The connection of LPA and its receptors to gene expression has become an interesting focus of research to understand the molecular mechanisms of LPA signal transduction.

Many biological effects of GPCR have been thought to occur through transactivation of EGFR [21,22]. In our previous studies, however, the effects of LPA on gene expression were much more potent than those of EGF or other agonists of receptor tyrosine kinases (RTKs) [15,19]. LPA indeed induced low levels of tyrosine phosphorylation of EGFR which were in no means comparable to that stimulated by EGF itself [19,23]. Intriguingly, the effect of LPA on its target gene Cox-2 was sensitive to inhibition of EGF, suggesting requirement of a permissive or parallel input from EGFR in the delivery of signals of LPA or other GPCR agonists [18,19]. In the current study, we explored the role of RTK in LPA activation of G protein signaling cascades and the downstream transcription factors. Molecular and pharmacological studies indicated that activation of the effectors of Gi, but not those of Gq or G12/13 relied on EGFR. Furthermore, activation of AP-1 components by LPA involved Gi signaling and was highly sensitive to inhibition of EGFR. We further demonstrated that this mode of crosstalk between GPCR and EGFR was mediated by the activity of a RTK, not necessarily EGFR. In contrast to AP-1, LPA stimulated another prominent transcription factor NF-κB via the Gq-PKC cascade in an EGFR-independent manner. These results demonstrate the involvement of EGFR or an alternate RTK in activation of selective G protein signaling cascades and the downstream responses.

## Methods

### Materials

1-oleoly (18:1) LPA and sphingosine 1 phosphate (S1P) were obtained from Avanti Polar Lipids, Inc. (Alabaster, AL). Prior to use, these phospholipids were dissolved in PBS containing 0.5% fatty acid-free bovine serum albumin (BSA). BSA, Fugene 6 and protease inhibitor cocktail tablets were purchased from Roche (Indianapolis, IN). Plasmid DNA was purified using the endo-free purification kit from Qiagen (Valencia, CA). Oligonucleotides and primers were synthesized by Operon Biotechnologies, Inc. (Huntsville, AL). Anti-phospho NF-κB p65 (Ser 536), anti-phospho IκBα (Ser 32), anti-phospho PKD (Ser 916), anti-phospho EGFR (Y 1068), anti-phospho tyrosine (Y-p), anti-Ras, and anti-tubulin α/β antibodies were obtained from Cell Signaling (Danvers, MA). Anti-EGFR antibody recognizing Ala351-Asp364 of the human EGF receptor was obtained from Millipore (Billerica, MA). Insulin, TRIzol and cell culture reagents were obtained from Invitrogen Inc. (Carlsbad, CA). Fetal bovine serum (FBS) was from Atlanta Biologicals (Lawrenceville, GA). Insulin-like growth factor (IGF) was obtained from Upstate Biotechnology (Lake Placid, NY). Hepatocyte growth factor (HGF) and the Quantikine IL-8 ELISA kit were obtained from R & D systems (Minneapolis, MN). Epidermal growth factor (EGF), platelet derived growth factor (PDGF), AG1478 and anti-β actin monoclonal antibody were obtained from Sigma (St. Louis, MO). Other antibodies used were from Santa Cruz Biotechnology (Santa Cruz, CA).

### Reporter vectors and luciferase assays

The AP-1 responsive reporter vector pGL2-3xAP-1-TK-Luc was constructed as described previously [15]. The NF-κB responsive luciferase vector p5xNF-κB-Luc was obtained from Stratagene (La Jolla, CA). Cells were seeded in 6-well plates and transfected with luciferase vectors using *trans*IT-TKO transfection reagent (Mirus Bio Corp., Madison, WI) according to the manufacturer. After 48 hours, the cells were starved for 24-36 hours before stimulation with 10 μM LPA or vehicle for 6 hours. Cell extracts assayed for luciferase activity using the luciferase assay kits from Promega. The luciferase activity was normalized on the basis of the activity of co-transfected β-galactosidase reporter driven by the cytomegalovirus promoter (pCMVβ-gal).

### Cell Culture

The sources and maintenance of ovarian cancer cell lines used in the study were described previously [15,19].

### Western blotting

Cells were lysed in SDS sample buffer or in ice-cold X-100 lysis buffer [1% Triton X-100, 50 mM HEPES (pH 7.4), 150 mM NaCl, 1.5 mM MgCl_2_, 1 mM EGTA, 10% glycerol, 100 mM NaF, 10 mM Na PPi, and protease inhibitor cocktail]. Total cellular proteins were resolved by SDS-PAGE, transferred to Immun-Blot membrane [poly(vinylidene difluoride)] (BIO-RAD, Hercules, CA), and immunoblotted with antibodies following the protocols of manufacturers. Immunocomplexes were visualized with an enhanced chemiluminescence detection kit from Amersham (Piscataway, NJ).

### Adenovirus and plasmids

The recombinant adenovirus carrying a truncated EGFR-CD533 lacking the 533 amino acids at the cytosolic domain was purified and used to infect cancer cell lines as described previously [24]. The truncated form of EGFR was also amplified by RT-PCR from Caov-3 cells using primers: EGFR-DN-Fwd 5' CATAAGCTTGG-AGCAGCGATGCGACCCTCC 3' and EGFR-DN-Rev 5' CATCTCGAGGCGCTTCCGAAC-GATGTGG3'. The fragment was cloned into the pcDNA3 expression vector. The Gq cDNA in pcDNA3 was kindly provided by Dr. RD Ye (University of Illinois at Chicago). The dominant-negative G208A mutant [25,26] was made by using the QuikChange XL site directed mutagenesis kit from Stratagene.

### Nuclear extracts and electromobility shift assay (EMSA)

LPA-stimulated or control cells were washed twice with cold PBS, harvested by scraping with a rubber policeman and centrifuged at 1000 rpm for 3 min with an Eppendorf microcentrifuge. Cell pellets were resuspended in a hypotonic lysis buffer [10 mM Tris-HCl (pH 7.4), 10 mM KCl, 3 mM MgCl_2_, 0.5% NP-40], incubated for 15 min on ice, and centrifuged at 3000 rpm. The pellets were washed once with the hypotonic lysis buffer, resuspended in hypertonic nuclear lysis buffer [50 mM Tris-HCl (pH 8.3), 0.4 M NaCl, 40% glycerol, 5 mM MgCl_2 _and 0.1 mM EDTA] and further incubated for 10 minutes before centrifugation at 13,000 rpm. The supernatants were collected and quick-frozen in liquid nitrogen before storage at -80°C. Protein concentrations were determined with the Pierce BCA kit.

AP-1 and NF-κB consensus oligonucleotides (AP-1 sense 5' GGCGCTTGATGACTCA-GCCGGAA 3'; AP-1 antisense 5'GGTTCCGGCTGAGTCATCAAGCG 3'; NF-κB sense 5'ATGTTGAGGGGACTTTCCCAGGCGG 3' and NF-κB antisense 5' GCCTGGGAAAGTC-CCCTCAACTGG 3') were annealed in 20 mM Tris (pH 7.4), 1 mM dithiothreitol, 50 mM NaCl and 10 mM MgCl_2_. Oligonucleotides were labeled by filling in at 3' end with [α-^32^P] dCTP using Klenow. The probe and protein binding was analyzed by incubating 4 μg of nuclear proteins in gel shift assay buffer [10 mM HEPES (pH 7.8), 10% glycerol, 1 mM EDTA, 25 mM MgCl_2_, 50 mM KCl, 1 μg of poly(dI.dC), 3 μg BSA and protease inhibitors] in a final volume of 20 μL for 10 min at 25°C. The binding specificity was confirmed by cold competition with 50-fold excess of unlabeled oligonucleotides. Complexes were separated by electrophoresis on 5% non-denaturing polyacrylamide gel (PAGE). Gels were dried under vacuum and subjected to autoradiography using a phosphoimager.

### Rho and Ras activation assays

Activation of Rho and Ras was analyzed by glutathione S-transferase (GST) pulldown assays [27,28]. The cells were grown in 10-cm dishes to subconfluency, starved overnight, and stimulated with LPA or vehicle. The cells were lysed in Magnesium-containing lysis buffer MLB (25 mM HEPES, pH 7.5, 150 mM NaCl, 1% NP40, 10% glycerol, 10 mM MgCl2, 1 mM EDTA, 1 mM sodium orthovanadate, 10 μg/ml leupeptin, 10 μg/ml aprotinin). Clarified lysates were incubated for 45-60 minutes at 4°C with GST-Rhotekin-RBD (Rho binding domain of the mouse Rhotekin, residues 7-89) [28] or GST-Raf-RBD (Ras binding domain of the human Raf, residues 1-149) [27] produced in Escherichia coli and immobilized onto glutathione-coupled Sepharose beads. Beads were washed in MLB three times, eluted with SDS sample buffer, and analyzed by immunoblotting.

### Migration and invasion assays

The migration of SKOV-3 cells was assayed using transwell chambers (pore size 8 μM) (BD Biosciences, Bedford, MA) as we described recently [29]. The inserts were precoated with type I collagen. Serum-starved cells were loaded to the upper chamber with or without AG1478. LPA was added to the lower chambers. Nonmigrated cells were removed from the top filter surface with a cotton swab. Migrated cells attached to the underside of the transwells were washed with PBS and stained with crystal violet and counted under a microscope. The invasion of SKOV-3 cells was measured using transwells coated with growth factor-reduced basement membrane matrix (pore size 8 μM) (BD Biosciences).

### Wound Closure Assay

Confluent monolayers of Caov-3 were serum starved for 18 hours. Scratches were made using sterile pipette tips. Displaced cell debris was washed off with serum-free media before stimulation with LPA or BSA with or without AG1478.

### Statistics

All numerical data were presented as mean ± SD. The statistical significance of differences was analyzed using Student's *t *test where *p *< 0.05 was considered statistically significant.

## Results

### Activation of AP-1 proteins by LPA, EGF or HGF

**Figure 1:**
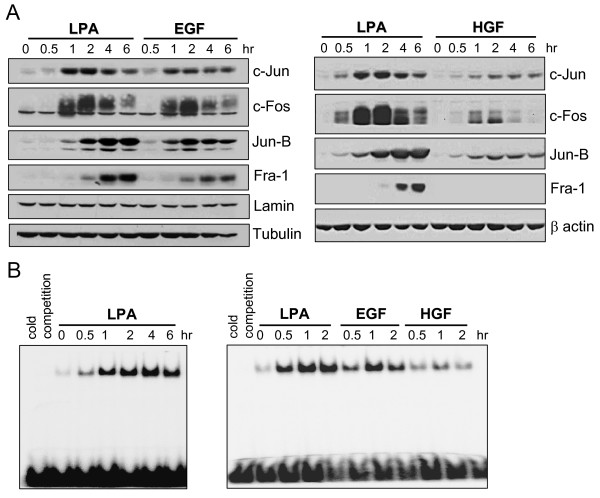
**Activation of AP-1 by LPA, EGF or HGF**. ***A***. Caov-3 cells were starved and stimulated for the indicated periods of time with LPA (10 μM), EGF (25 ng/ml) or HGF (20 ng/ml). The cell lysates were analyzed by immunoblotting for expression of AP-1 proteins c-Jun, c-Fos, Jun-B and Fra-1 and other loading controls (lamin A/C, β actin or tubulin). ***B***. Caov-3 cells were stimulated as in ***A ***and analyzed for AP-1 DNA binding activity with EMSA by incubating with ^32^P-labeled AP-1 consensus oligonucleotides. Reaction mixes were run on 5% native polyacrylamide gels and autoradiographed. The specificity of binding to the ^32^P-labeled AP-1 probe in nuclear extracts of cells treated with LPA for 2 hours was confirmed by inhibition of the binding with 50 times of unlabeled oligonucleotides (cold competition).

LPA is a master inter-cellular regulator of gene expression in mammalian cells, especially in human cancer cells that express multiple LPA receptor subtypes [15,17,19,20,30]. Although post-transcriptional regulation may be involved in the reinforcement of the LPA's effect on gene expression [19], the major activity seems to be afforded by transcriptional upregulation. We have shown that a number of transcription factors such as C/EBP, NF-κB, AP-1, c-Myc and Sp-1 individually or coordinately initiate transcription of LPA target genes [15,19,20]. Thus analysis of transcriptional activation offers an ideal readout to study functionality of LPA receptors, their downstream signaling networks and their crosstalk with RTKs. In ovarian and other cancer cell lines, treatment with LPA led to induction of multiple AP-1 proteins. As demonstrated in Fig. 1A, LPA induced c-Jun, Jun-B, c-Fos, and Fra-1 proteins in a time-dependent manner in Caov-3 cells. LPA also triggered phosphorylation of c-Jun and c-Fos (data not shown). Induction of c-Jun and c-Fos expression peaked at 1-2 hours after exposure to LPA. Jun-B and Fra-1 were induced later and the highest levels were observed at 4-6 hours of LPA treatment (Fig. 1A).

The sequential induction of these AP-1 components could lead to sustained increases in AP-1 activity. Indeed, EMSA confirmed elevation in AP-1 DNA-binding activity in LPA-treated cells, which lasted for many hours (Fig. 1B). Consistent with early induction of c-Jun and c-Fos and delayed induction of Jun-B and Fra-1, AP-1 DNA-binding activity was increased at 0.5 hour, reached plateau at 1 hour and remained at the highest levels up to 6 hours after addition of LPA. We further confirmed that LPA treatment resulted in transcriptional activation of AP-1. Caov-3 cells were transfected with the AP-1 responsive luciferase reporter pGL2-3xAP-1-TK-Luc. Treatment with 10 μM LPA for 6 hours induced more than 25-fold increases in luciferase activity compared to vehicle-treated cells (see Fig. 3C).

**Figure 2:**
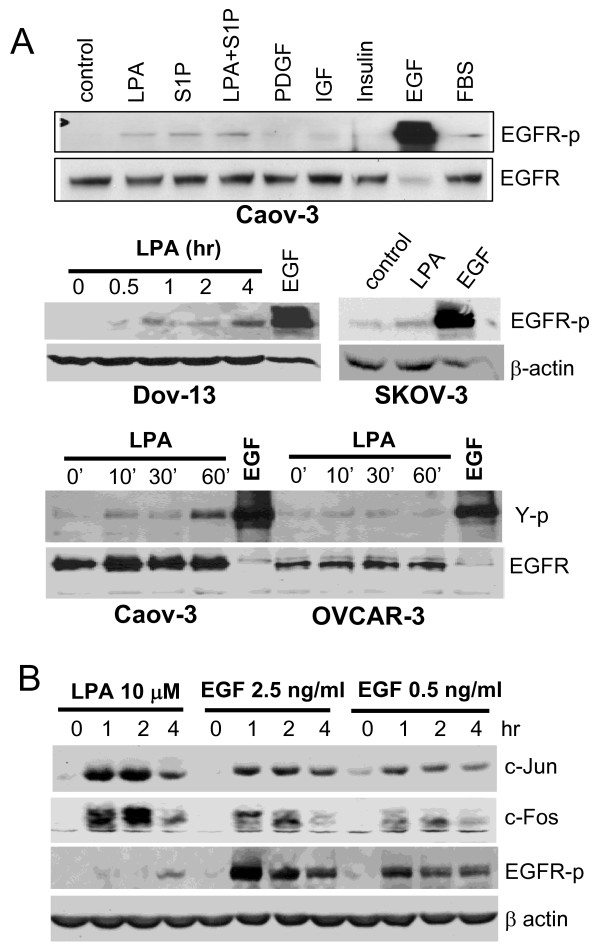
**Modest increase in tyrosine phosphorylation of EGFR induced by LPA**. ***A***. Caov-3, Dov-13 and SKOV-3 cells were staved and stimulated for 30 min or otherwise indicated (hr) with LPA (10 μM), S1P (1 μM), LPA (10 μM)+S1P (1 μM) IGF (50 ng/ml), insulin (0.2 μM), PDGF (BB isoform, 50 ng/ml), EGF (25 ng/ml) or FBS (5%). Tyrosine phosphorylation of EGFR was examined by immunoblotting with an anti EGFR phospho-specific antibody that recognizes EGFR-p at Y1068. The membrane was reprobed for total EGFR or β actin. In the *bottom panel*, Caov-3 and OVCAR-3 cells were treated with LPA (10 μM) for the indicated periods of time (min) or with EGF (25 ng/ml) for 30 min as a positive control. EGFR was immunoprecipitated from cell lysates and then analyzed by immunoblotting with an anti phosphotyrosine antibody for tyrosine phosphorylation (Y-p). ***B***. Caov-3 cells were treated with LPA or different doses of EGF as indicated. Induction of AP-1 proteins and EGFR-p was examined by immunoblotting.

In these experiments, we also analyzed EGF and HGF for their ability to activate AP-1. The effects of LPA on AP-1 protein expression and AP-1 DNA-binding activity were stronger or at least comparable to those of EGF (Fig. 1). Since LPA induced only nominal EGFR activation as reflected by a modest increase in EGFR phosphorylation at Y1068 or in overall tyrosine phosphorylation of EGFR as surrogate measurement of EGFR activation (Fig. 2), it is unlikely that LPA stimulated AP-1 through transactivated EGFR. Compared to HGF, LPA was much more efficacious in inducing each of AP-1 proteins (Fig. 1A) and in increasing AP-1 DNA-binding activity (Fig. 1B). For example, HGF only modestly stimulated expression of c-Jun, c-Fos, Jun-B and failed to induce Fra-1 (Fig. 1A). Accordingly, only a weak and transient increase in AP-1 DNA-binding activity was detected in HGF-treated cells compared to that seen in LPA-stimulated cells (Fig. 1B).

### Requirement of EGFR or an alternate RTK for LPA activation of AP-1

**Figure 3:**
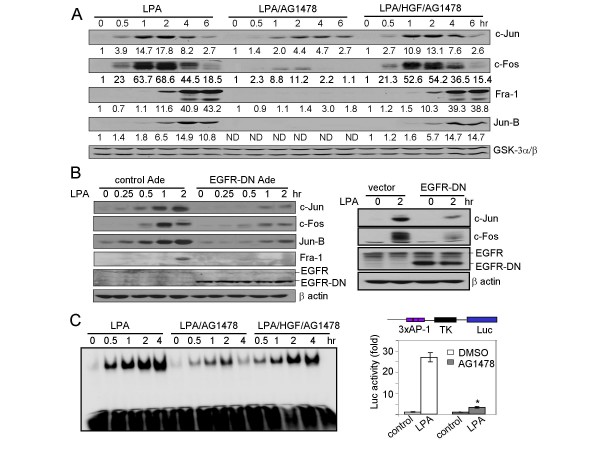
**Requirement of EGFR or an alternate receptor tyrosine kinase for LPA-induced activation of AP-1**. ***A***. LPA-induced expression of AP-1 proteins was suppressed by the EGFR inhibitor AG1478, an effect reversible by costimulation with HGF. Caov-3 cells were starved and stimulated with LPA in the presence of vehicle, AG1478 (LPA/AG1478), or AG1478 and HGF (LPA/HGF/AG1478). AG1478 (1 μM) was added 45 min before stimulation with LPA (10 μM) or LPA plus HGF (20 ng/ml). AP-1 proteins and the loading control GSK-3α/βwere analyzed by immunoblotting. The results were quantified by densitometry with values of the un-stimulated, control cells in each set of treatment defined as 1 fold. ND refers to signals not detectable. Similar results were obtained from two independent experiments. ***B***. LPA-induced expression of AP-1 proteins was also inhibited by a dominant negative mutant of EGFR (EGFR-DN). EGFR-DN was expressed in Caov-3 cells via adenovirus (*left panel*) or transient transfection (*right panel*). The recipient cells were starved and stimulated for the indicated periods of time with 10 μM LPA followed by immunoblotting for expression of AP-1 proteins, phosphorylated p65, and EGFR-DN. ***C***. LPA-induced AP-1 DNA-binding activity was dependent on EGFR or an alternate RTK. Nuclear extracts were prepared from Caov-3 cells treated for the indicated periods of time with LPA in the presence of vehicle (DMSO), AG1478, or AG1478 plus HGF as described in ***A***. The AP-1 DNA-binding activity of nuclear extracts was analyzed with EMSA. ***D***. The AP-1 transcriptional activity was sensitive to AG1478. Caov-3 cells were transfected with the AP-1 reporter vector pGL2-3xAP1-TK-Luc. The cells were starved and stimulated for 6 hours with LPA (10 μM) in the presence of AG1478 (1 μM) or vehicle. Luciferase activity in cell lysates was determined with luciferase assay kits and the results expressed as fold induction with the basal activity in unstimulated control cells defined as 1 fold. The statistical significance of differences of data in this and following figures was determined by Student's t test, where *p *< 0.05 was considered statistically significant and marked with an asterisk.

Transactivation of EGFR does not seem to account for the dramatic stimulation of AP-1 activity by LPA (Fig. 2). However, pretreatment of Caov-3 cells with AG1478, a specific pharmacological inhibitor of EGFR kinase activity [31] abrogated expression of Jun-B and Fra-1 and dramatically decreased c-Fos and c-Jun expression in LPA-treated cells (Fig. 3A). In agreement with the inhibition of AP-1 protein expression, AG1478 also dramatically suppressed LPA-stimulated AP-1 DNA-binding and transcriptional activities in Caov-3 cells (Fig. 3C &3D). Similar results were obtained from the SKOV-3 and Dov-13 ovarian cancer cell lines (data not shown).

To confirm that AG1478 indeed specifically inhibited EGFR instead of toxic or non-specific interference with other targets, we expressed a truncated form of EGFR (EGFR-CD533) lacking the cytosolic domain and thereby functioning as a dominant negative mutant (EGFR-DN) through dimerization with wild type EGFR [24]. EGFR-DN was introduced into Caov-3 cells using adenovirus expressing EGFR-DN [24] or by transient transfection of pcDNA3-EGFR-DN with Amaxa nuocleofector Kit T that yield high transfection efficiency in ovarian cancer cell lines as we described previously [19]. As shown in Fig. 3B, expression of EGFR-DN indeed inhibited LPA-induced expression of AP-1 proteins. These results demonstrated that an intact EGFR is indispensable for LPA activation of AP-1.

To address whether EGFR, instead of other RTKs, is specifically required for GPCR signaling to AP-1, we co-stimulated Caov-3 cells with LPA and HGF, an agonist of c-Met, in the presence of the EGFR blocker AG1478. As shown in Fig. 3A, the inhibitory effect of AG1478 on AP-1 proteins was reversed by co-stimulation of the cells with LPA and HGF. LPA-induced AP-1 DNA binding activity was also restored by HGF in the presence of AG1478 (Fig. 3C). The impact of HGF was not due to activation of AP-1 proteins by HGF itself as the effect of HGF on AP-1 was marginal compared to that of LPA (Fig. 1A &1B). These results indicate that the activity of a RTK, not necessarily EGFR, provides a permissive input to allow transmission of GPCR signals to AP-1 although such an input itself is not sufficient to induce full AP-1 activation.

### EGFR-independent activation of NF-κB by LPA

**Figure 4:**
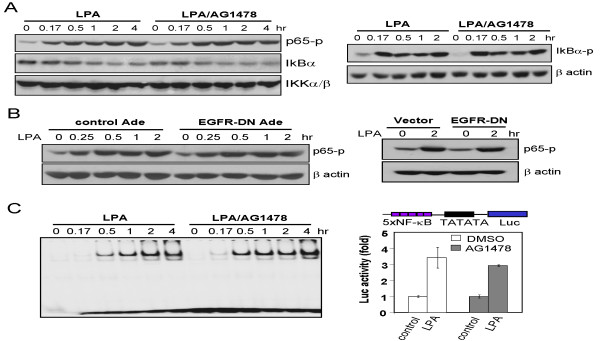
**EGFR-independent activation of NF-κB by LPA**. ***A***. LPA-induced NF-κB activation was resistant to AG1478. Caov-3 cells were starved and stimulated for the indicated periods of time with LPA (10 μM) in the presence of AG1478 (1 μM) or vehicle (DMSO). AG1478 was added 45 min before stimulation with LPA. NF-κB p65 phosphorylation at Ser 536, IκBα degradation and total IKKα/β (*left panel*), and IκBα phosphorylation at Ser 32 (*right panel*) were analyzed by immunoblotting. ***B***. LPA-induced NF-κB activation was refractory to expression of EGFR-DN. EGFR-DN was introduced into Caov-3 cells via recombinant adenovirus (*left panel*) or transient transfection (*right panel*) and expressed as shown in Fig. 3B. LPA-induced p65 phosphorylation was analyzed by immunoblotting. ***C***. LPA-induced NF-κB DNA-binding and transcriptional activities were unaffected by inhibition of EGFR. Nuclear extracts were prepared from Caov-3 cells stimulated for the indicated periods of time with LPA (10 μM) in the presence of AG1478 or vehicle as in ***A***. The NF-κB DNA binding activity was determined with EMSA (*left panel*). LPA-induced NF-κB transcriptional activity was analyzed by luciferase assay (*right panel*). The cells were transfected with p5xNF-κB-Luc, starved and stimulated for 6 hours with LPA (10 μM) in the presence of AG1478 (1 μM) or vehicle. The results were presented as fold induction with the basal activity in unstimulated cells defined as 1 fold.

The role of EGFR in LPA induction of AP-1 activity raises the possibility that EGFR might be required ubiquitously for GPCR actions. This could be due to the requirement of a RTK activity for overall functioning of GPCR. However, if the RTK input is implicated in activation of the specific intracellular signaling processes instead of GPCR itself, certain LPA signaling pathways may be exceptional to this requirement. To distinguish these possibilities, we examined LPA-induced activation of NF-κB, another prominent transcription factor critically involved in activation of many LPA target genes [15]. In Caov-3 treated with AG1478 or overexpressing EGFR-DN, LPA induced NF-κB p65 phosphorylation (Ser 536), IκBα phosphorylation (Ser 32) and IκBα degradation at levels comparable to those detected in control cells with intact EGFR (Fig. 4A &4B). Similarly, LPA-stimulated NF-κB DNA-binding activity was not compromised by AG1478 as measured by EMSA (Fig. 4C). Nor was LPA-driven NF-κB transcriptional activity significantly affected by incubation of cells with AG1478 as analyzed by the NF-κB responsive luciferase reporter assay (Fig. 4C). Therefore, in sharp contrast to AP-1 upregulation, LPA-induced NF-κB activation occurs via an EGFR-independent route. The results also indicate that the crosstalk with RTK is required only for a selective subset of biochemical events downstream of LPA receptors but not ubiquitous receptor activation.

### G protein cascades mediating AP-1 and NF-κB activation

**Figure 5:**
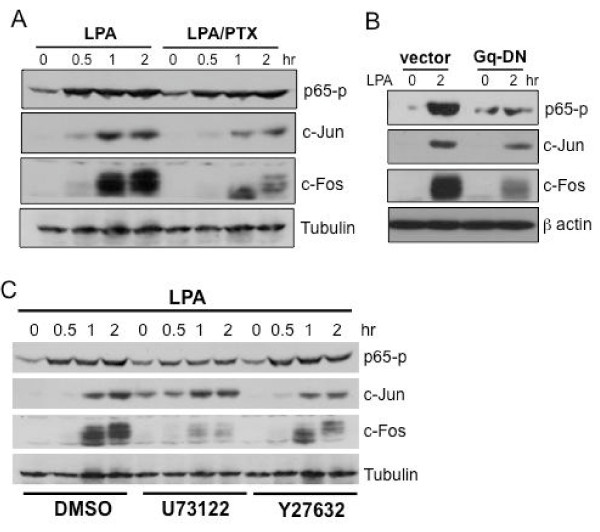
**Involvement of different G protein signaling cascades in LPA-induced activation of AP-1 and NF-κB**. ***A***. Inhibition of Gi with PTX attenuated AP-1 but not NF-κB activation by LPA. Caov-3 cells were starved and stimulated for the indicated periods of time with LPA (10 μM) in the presence or absence of PTX (25 ng/ml). PTX was added 6 hours before stimulation with LPA. LPA-induced phosphorylation of NF-κB p65 at Ser 536 and expression of c-Jun and c-Fos were analyzed by immunoblotting. ***B***. Caov-3 cells were transfected using Amaxa with the Gq G208A dominant negative mutant (Gq-DN) or control vector. The transfected cells were starved and stimulated for 2 hr with LPA (10 μM) before immunoblotting for p65 phosphorylation and c-Jun and c-Fos expression. ***C***. Caov-3 cells were starved and stimulated for indicated periods of time with LPA (10 μM) in the presence or absence of U73122 (10 μM) or Y27632 (7.5 μM) which were added 45 min before stimulation with LPA. NF-κB p65 phosphorylation and c-Jun and c-Fos expression were assessed by immunoblotting. Reprobing with tubulin or β actin was included as loading controls.

To identify the mechanism for the differential requirements of RTK in the delivery GPCR signals to AP-1 and NF-κB, we examined G protein cascades regulating AP-1 and NF-κB activation. The classical Edg LPA receptors expressed in cancer cell lines couple to Gi, Gq and G12/13 [4-7]. Pertussis toxin (PTX), a selective inhibitor of Gi proteins, strongly decreased LPA-induced AP-1 proteins c-Jun and c-Fos as shown in Fig. 5A, indicating that the Gi signaling links to AP-1 activation by LPA. However, Gi was dispensable for NF-κB activation as PTX did not alter NF-κB p65 phosphorylation induced by LPA in these cells (Fig. 5A).

To assess the contribution of Gq signaling to AP-1 and NF-κB activation, we used a dominant negative form of Gq (G208A) that has been shown to specifically block Gq-mediated pathways in different cell systems [25,26]. The Gq-DN mutant (pcDNA3-GqG208A) was transfected into Caov-3 cells using Amaxa. Expression of the Gq mutant almost completely prevented LPA-induced NF-κB p65 phosphorylation (Fig. 5B). It also strongly decreased c-Fos expression and slightly decreased c-Jun induction in response to LPA (Fig. 5B). Because of the lack of commercially available inhibitor of Gq, these effects on c-Jun, c-Fos and NF-κB p65 of GqG208A were further confirmed by using U73122 [32], an inhibitor of phospholipase C that lies downstream of Gq (Fig. 5C). Therefore, the Gq-mediated signaling is critical for LPA stimulation of NF-κB and contributes to LPA induction of the AP-1 component c-Fos.

We also examined the role of G12/13 in LPA-mediated activation of AP-1 and NF-κB by inhibition of the G12/13 effector ROCK. ROCK has been reported to participate in LPA-induced c-Jun expression in NIH 3T3 cells [33]. We examined the effect of the specific ROCK inhibitor Y27632 on LPA-induced AP-1 and NF-κB activation. The compound did not affect LPA-induced p65 phosphorylation but compromised c-Jun and c-Fos induction (Fig. 5C). Based on these results, each of G protein modules (Gi, Gq, and G12/13) seems to contribute to AP-1 activation but only Gq couples to the NF-κB activation in LPA-stimulated cells.

### Differential requirement of EGFR for activation of G protein cascades

**Figure 6:**
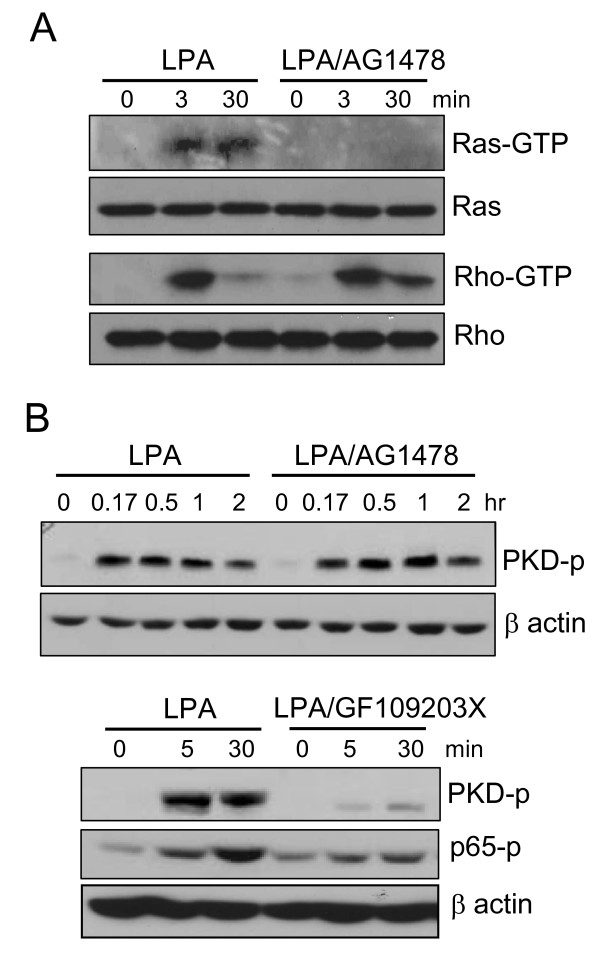
**Differential requirement of EGFR for activation of G protein signaling cascades by LPA**. ***A***. Inhibition of EGFR blocked LPA-stimulated Ras but not Rho activation. Caov-3 cells were starved and stimulated for the indicated periods of time with LPA (5 μM) in the presence of AG1478 (1 μM) or vehicle (DMSO). The levels of Ras-GTP and Rho-GTP were determined by pulldown with GST-Raf and GST-Rhotekin, respectively as described in Methods. ***B***. Inhibition of EGFR did not affect signaling of the Gq-PKC pathway. Caov-3 cells were starved and stimulated for the indicated periods of time with LPA (10 μM) in the presence of AG1478 (1 μM), GF109203X (2.5 μM) or vehicle. LPA-induced phosphorylation of PKD at Ser 916 and NF-κB p65 at Ser 536 was analyzed by immunoblotting followed by reprobing with β actin.

We next explored whether EGFR is differentially required for activation of the diverse G protein signaling modules. Since it is practically difficult to quantitate activation of different classes of G proteins, we measured the activation of the downstream effectors of G proteins. Specifically, Ras is a well-defined Gi-dependent signal [4,12]. Rho activation lies downstream of G12/13 [5,12]. Gq activation could be monitored by analyzing the downstream PKC-PKD pathway [34]. GST pulldown assays were employed to analyze LPA-triggered Ras and Rho activation [27,28]. As demonstrated in Fig. 6A, AG1478 abolished LPA-induced Ras activation as suggested by loss of GTP-bound Ras (GTP-Ras) in LPA-stimulated cells. In agreement with EGFR-dependent activation of Ras by LPA, Gi-linked Erk activation was also inhibited by AG1478 (data not shown), suggesting that Gi activation by LPA relies on a permissive signal from EGFR. However, the presence of AG1478 did not interfere with LPA-induced increases in Rho-GTP (Fig. 6A), indicating that EGFR activity is not essential for activation of the Rho-ROCK pathway. Similarly, inhibition of EGFR with AG1478 did not impair signal transmission from Gq to PKD as reflected by the retention of a full magnitude of PKD phosphorylation (Ser 916) induced by LPA (Fig. 6B). LPA-stimulated PKD phosphorylation was suppressed by the PKC inhibitor GF 109203X (Fig. 6B), which also attenuated NF-κB p65 phosphorylation in response to LPA (Fig. 6B). Therefore, activation of the Gq-PLC-PKC pathway and the downstream NF-κB by LPA does not require EGFR. These results reveal EGFR-dependent Gi and EGFR-independent Gq and G12/13 signaling cascades downstream of LPA receptors. Since these G protein cascades are coupled to specific transcription factors as identified above, the results provide a molecular basis for the differential requirement of EGFR in LPA activation of AP-1 and NF-κB.

### Roles of EGFR in multiple biological responses to LPA

**Figure 7:**
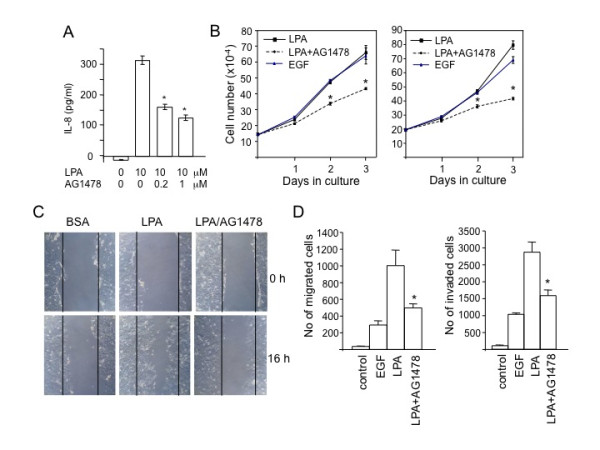
**Participation of EGFR in LPA-induced IL-8 production, cell proliferation, migration and invasion**. ***A***. Caov-3 cells cultured in 6-well plates were starved and stimulated for 16 hours with LPA (10 μM) in the presence of indicated concentrations of AG1478. Levels of IL-8 released to culture supernatants were analyzed by ELISA. ***B***. Caov-3 and SKOV-3 cells were cultured in 6-well plates with serum-free medium supplemented with EGF (25 ng/ml) or with 5 μM LPA in the presence of AG1478 (1 μM) or vehicle for 1-3 days. The cell numbers from triplicate wells were determined daily with a coulter counter. ***C***. Caov-3 cells were grown in 60 mm dishes to confluence and starved before scratches were made with sterile pipettes. The cells were incubated for 16 hours with BSA or LPA (5 μM) in the presence of AG1478 (1 μM) or vehicle. Microscopic images (76×) were captured at 0 and 16 hours after LPA exposure. ***D***. The migration and invasion of SKOV-3 cells in response to EGF (25 ng/ml) or to LPA (1 μM) in the absence or presence of AG1478 (1 μM) were analyzed using transwells and transwells coated with growth factor-reduced basement membrane matrix, respectively. SKOV-3 cells were starved with or without AG1478 before trypsinization. The cells (2.5 × 10^4 ^cells/0.5 ml for migration and 1 × 10^5^/0.5 ml for invasion) were loaded to the upper chambers and allowed to migrate for 6 hours or invade for 20 hours. The migrated and invaded cells on the underside of transwells were stained with crystal violet and counted under microscope. Results were average numbers of migrated or invaded cells/transwell from triplicates (mean+ SD). The data shown was representative of three independent experiments.

If Gi and the downstream AP-1 depend on an EGFR permissive signal for activation, we expected that many cellular processes mediated by Gi or AP-1 would be sensitive to inhibition of EGFR. To further test this speculation, we examined the effect of EGFR inhibition on LPA-induced IL-8 production, a functional outcome of synergistic activation of NF-κB and AP-1 as we described previously [15]. Although EGF its own only weakly stimulated IL-8 generation [15], the prominent effect of LPA was suppressed by inhibition of EGFR with AG1478 (Fig. 7A). The data was consistent with participation of EGFR in LPA-induced AP-1 activation and the subsequent IL-8 production.

Due to the crucial role of Gi-mediated signals in promotion of cell proliferation and motility [12,29,35], one would predict that these biological responses to LPA should be also attenuated by inhibition of EGFR. Indeed, as shown in Fig. 7B, AG1478 significantly decreased LPA-stimulated cell growth in Caov-3 and SKOV-3 cells. AG1478 also inhibited LPA-induced migration and invasion of these cells as assessed in transwell chambers in SKOV-3 cells or by wound healing assay in Caov-3 cells (Fig. 7C &7D).

## Discussion

We have previously shown that LPA induces transcriptional activation of multiple cancer-associated genes [15,17,19,20]. In these studies, ovarian cancer cells were used as a model system that express multiple LPA receptor subtypes and respond robustly to physiological levels of LPA. We demonstrated that LPA drives gene expression via a broad range of transcription factors [15,17,19,20]. However, the signaling processes linking LPA to transcriptional activation remain poorly understood. In the current study, we have focused on the crosstalk between LPA receptors and EGFR in G protein signaling to transcription factors. Our results indicate that LPA-induced activation of AP-1 relies on a permissive activity from EGFR while LPA stimulates NF-κB in an EGFR-independent manner. The differential requirements of EGFR for AP-1 and for NF-κB suggest that the EGFR signal is involved in activation of a subset of intracellular signaling cascades of LPA receptors rather than the overall functionality of LPA receptors. Furthermore, we identified EGFR-dependent and independent G protein signaling cascades implicated in activation of the AP-1 and NF-κB transcription factors. Our results establish that the Gi-mediated pathway relies on EGFR for activation while Gq and G12/13 signals are refractory to inhibition of EGFR. AP-1 activation by LPA involves signaling of Gi, Gq, and G12/13 and therefore is EGFR dependent. On the other hand, activation of NF-κB by LPA is mediated through an EGFR-independent Gq signaling process with little contribution from Gi or G12/13 pathway.

The crosstalk between RTK and GPCR in cellular functions has been a subject of extensive research in the area of signal transduction. The "transactivation" model has been proposed to explain the functional dependence of GPCR signals on RTK [21,22]. In contrast, the possibility for integration of RTK activity into specific signaling events of GPCR has been suggested but poorly studied [19,36]. In ovarian cancer cell lines challenged with LPA, we observed only weak transactivation of EGFR (Fig. 2). In addition, the effects of LPA on activation of transcription factors and the downstream gene expression were more profound than those of EGF [15,17,19]. Therefore, it is hard to imagine that LPA induces these biochemical and biological changes purely through transactivated EGFR. In contrast, our results are in concert with a permissive role of EGFR in activation of a subset of GPCR signals. Elucidation of EGFR-dependent and EGFR-independent G protein signaling cascades and their downstream biochemical events allow us to conclude that only selective GPCR signaling pathways are regulated by EGFR.

It remains to be determined how EGFR is integrated with GPCR signaling to activate Gi and the downstream processes. The EGFR signal may feed in at Gi or at some points of the Gi axis. EGFR may be required for tyrosine phosphorylation of Gi or another component of the Gi pathway. Since the role of EGFR could be substituted for by activation of another RTK such as c-Met, it is unlikely that EGFR physically interacts with LPA receptors to facilitate Gi activation. Most likely, a RTK activity, not necessarily EGFR, catalyzes critical tyrosine phosphorylation of Gi or a Gi effector protein.

Compared to other RTKs, EGFR is more universally expressed and exhibits higher activity, particularly in cancer cells [37]. EGFR is amplified, overexpressed or activated through mutation in many types of human cancers including ovarian cancer [37,38]. Therefore EGFR is a well-recognized anti-cancer therapeutic target [37,38]. The elevated EGFR activity in malignant cells likely acts as a default RTK to crosstalk with GPCR. The basal, unstimulated activities of other RTKs may not be sufficient to serve such a role. However, when activated by their specific ligands, other RTKs such as c-Met can replace EGFR to provide a tyrosine kinase activity to allow GPCR signaling to Gi and the downstream networks. Based on our results presented in the current study, EGFR is essential for many biological processes evoked by GPCRs including cytokine production, cell proliferation, and cell migration and invasion. Diverse GPCR agonists are important mediators of cancer initiation and progression. Inhibition of EGFR in cancer patients may provide therapeutic benefits through not only disconnecting EGFR to its own direct effectors but also interfering with GPCR signaling.

## Conclusions

We have demonstrated that EGFR is required for activation of the AP-1 transcription factor and other Gi-dependent cellular responses to LPA. In contrast, activation of G12/13, Gq and Gq-elicited NF-κB by LPA is independent of EGFR signaling. This selective requirement of EGFR reflects engagement of a permissive signal from a RTK, not necessarily EGFR, in LPA activation of a subset of G protein cascades. These results provide a novel insight into the role of RTK in GPCR signal transduction and biological functions.

## List of Abbreviations Used

LPA: lysophosphatidic acid; EGFR: epidermal growth factor receptor; RTK: receptor tyrosine kinase; GPCR: G protein-coupled receptor; AP-1: activator protein 1; NF-κB: nuclear factor kappa B; PKC: protein kinase C.

## Statement Of Competing interests

The authors declare that they have no competing interests.

## Authors' contributions

RO and XF were responsible for experimental design and preparation of the manuscript. RO, SG, DD, AM, JW, and XF performed experiments. PD and ZL generated some experimental reagents. All authors read and approved the final version of the manuscript.
